# Solubility design leading to high figure of merit in low-cost Ce-CoSb_3_ skutterudites

**DOI:** 10.1038/ncomms8584

**Published:** 2015-07-20

**Authors:** Yinglu Tang, Riley Hanus, Sinn-wen Chen, G. Jeffrey Snyder

**Affiliations:** 1 Department of Applied Physics and Materials Science, California Institute of Technology, Pasadena, California 91125, USA; 2 Department of Materials Science and Engineering, Northwestern University, Evanston, Illinois 60208, USA; 3 Department of Chemical Engineering, National Tsing Hua University, #101, Sec.2, Kuang-Fu Rd., Hsin-Chu 300, Taiwan

## Abstract

CoSb_3_-based filled skutterudite has emerged as one of the most viable candidates for thermoelectric applications in automotive industry. However, the scale-up commercialization of such materials is still a challenge due to the scarcity and cost of constituent elements. Here we study Ce, the most earth abundant and low-cost rare earth element as a single-filling element and demonstrate that, by solubility design using a phase diagram approach, the filling fraction limit (FFL) *x* in Ce_*x*_Co_4_Sb_12_ can be increased more than twice the amount reported previously (*x*=0.09). This ultra-high FFL (*x*=0.20) enables the optimization of carrier concentration such that no additional filling elements are needed to produce a state of the art *n*-type skutterudite material with a *zT* value of 1.3 at 850 K before nano-structuring. The earth abundance and low cost of Ce would potentially facilitate a widespread application of skutterudites.

## Abstract (web summary)

Thermoelectric materials have the potential to convert waste heat into electricity. Although some of the more viable thermoelectric materials are based on expensive rare earth elements, here the authors replace Yb with low-cost Ce by engineering Ce solubility, thereby making Ce-CoSb3 a competitive thermoelectric.

## Graphical abstract

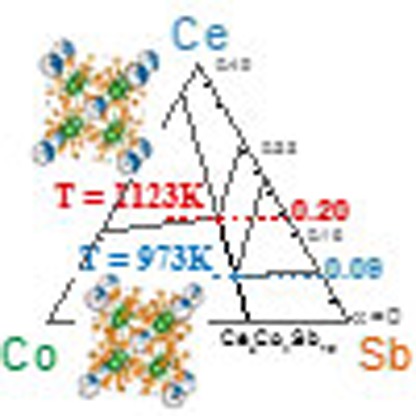

The world energy consumption has been increasing steadily and much effort is required worldwide to meet the challenging demand. Thermoelectric materials, which can convert waste heat into electricity, may help to reduce fossil fuel consumption and augment energy efficiency, and thus play an important role in the solution for the world's energy dilemma. The thermoelectric performance of materials is characterized by the dimensionless figure of merit, *zT*=*S*^*2*^*σT/κ*, where *S* is the Seebeck coefficient, *σ* is the electrical conductivity, *T* is the absolute temperature and *κ* is the total thermal conductivity.

Among various kinds of state-of-the-art thermoelectric materials, filled skutterudites distinguish themselves by allowing low thermal conductivity like a glass and high electrical conductivity like a crystal at the same time, which make them excellent thermoelectric materials according to the ‘phonon glass electron crystal' concept^1^. Moreover, the intermediate temperature range (400–600 °C) at which filled skutterudites have optimum thermoelectric performance makes this material a perfect candidate for automotive industry applications. Because of the heavy mass and small radius of the Yb atom compared with other fillers, Yb-CoSb_3_ skutterudites have excellent thermoelectric properties (*zT*=1.3 at 850 K for Yb_0.3_Co_4_Sb_12_) ref. 2 and have been selected by many automotive companies, such as General Motors, Ford and BMW, as the most promising thermoelectric material for waste heat recovery^3^. However, the deficiency of the rare earth element Yb (abundance in earth's crust 3 p.p.m.) could potentially be a problem in large-scale commercialization. Unlike Yb, Ce is the most earth abundant element of the rare earths (abundance in earth's crust 68 p.p.m.)^4^. In fact, there are huge resources of cerium because it is more abundant than other commonly known elements such as lead (14 p.p.m.), tin (2 p.p.m.), silver (0.07 p.p.m.), tellurium (0.005 p.p.m.) and gold (0.001 p.p.m.). The price of cerium is also more competitive compared with ytterbium. For large quantities, the difference is a factor of 10 (Ce; $5,000 per ton, Yb; $50,000 per ton^5^), placing Ce in the category of other commonly used elements such as Ni.

With its larger earth abundance and fairly low cost, use of Ce would help the commercialization of *n*-type skutterudites for automotive industry only if the thermoelectric properties of Ce-doped skutterudites can match those of Yb-doped skutterudites.

Interestingly, single-filled Ce-CoSb_3_ skutterudites have been considered as unsuitable thermoelectric materials and there has yet been little report of high temperature thermoelectric properties of pure Ce-CoSb_3_ skutterudites. Ce has been considered to be a good auxiliary filler in multiple-filled skutterudites, such as in (In, Ce)-CoSb_3_ skutterudites (*zT* of 1.43 for In_0.2_Ce_0.15_Co_4_Sb_12_ at 800 K)^6^ or (Ba, Ce)-CoSb_3_ skutterudites (*zT* of 1.26 for Ba_0.18_Ce_0.05_Co_4_Sb_12.02_ at 850 K)^7^. Ce has not typically been considered as a single filler. This is most likely due to its reported small filling fraction limit *x*=0.09 in Ce_*x*_Co_4_Sb_12_ (ref. 8) that would prevent Ce-containing skutterudites to achieve the optimum carrier concentration for *n*-type skutterudites^9^. To increase the filling fraction limit of Ce in CoSb_3_ skutterudites, charge-compensational doping, such as Fe or Mn-doping has been largely applied, which can dope the material from *n*-type to *p*-type^10^,^11^,^12^,^13^,^14^.

In this study, single-filling Ce-CoSb_3_ skutterudites without charge-compensational doping are prepared under equilibrium conditions and their thermoelectric properties are measured up to 850 K. The filling fraction limit of Ce is studied by solubility design using an equilibrium phase diagram approach. This approach introduces both the annealing temperature and nominal composition as variables in determining the filling fraction limit, which leads to the possibility of enhancing filling fraction of fillers thermodynamically rather than consider it as a single value. Using this phase diagram approach, a temperature-dependent solubility like the Yb in Yb-CoSb_3_ sheds light on optimizing Ce-CoSb_3_, whose thermoelectric properties are limited by its low filling fraction limit^2^. With solubility design, a clear solubility enhancement with a filling fraction limit of *x*=0.20 is discovered at 850 K for Ce-CoSb_3_ skutterudites. The surprisingly high filling fraction limit at high annealing temperatures enables the Ce-CoSb_3_ skutterudites to be optimized with *zT* of 1.3 for Ce_0.14_Co_4_Sb_12_ at 850 K, which makes it an excellent substitute for Yb-doped skutterudites for waste heat recovery applications.

## Results

### Optimum doping of Ce-CoSb_3_ skutterudites

**Figure 1:**
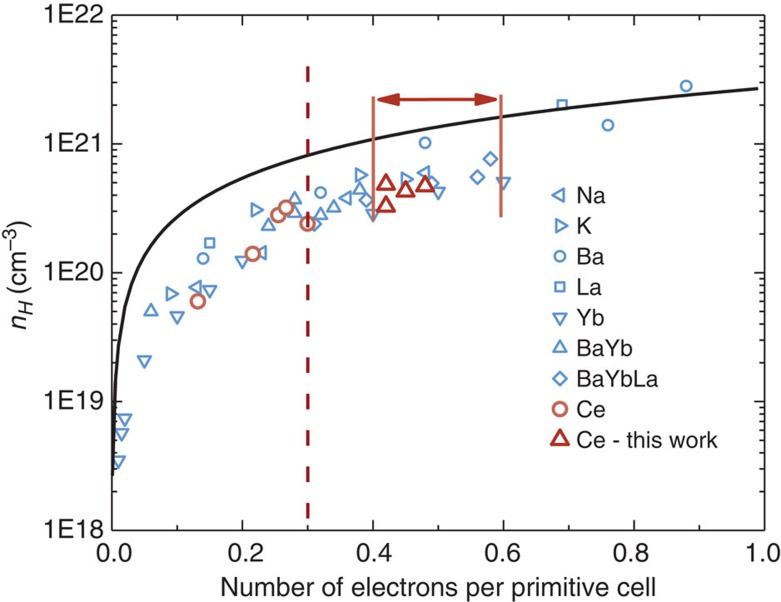
Hall carrier concentration versus number of electrons per primitive cell Co_4_Sb_12_. Data from refs 2, 8, 9, 18, 19, 32, 33, 34, 35. The number of electrons per primitive cell is calculated as the sum of *q*_i_*x_i_ for partially filled (*R*_1_)_*x*1_(*R*_2_)_*x*2…_(*R*_*n*_)_*xn*_Co_4_Sb_12_, where *q*_i_ is the effective charge state and *x*_i_ is the filling fraction of the ith filler *R* in multiple-filled skutterudites. The solid line represents the theoretical curve calculated by using *n*=2*x*/*a*^3^, where *x* is the total number of electrons per primitive cell and *a* is the lattice constant of CoSb_3_. The red double arrow represents the estimated high power factor region (see discussions below) and the red dashed line represents the number of electrons per primitive cell limited by the previously thought Ce filling fraction limit^8^.

**Figure 2:**
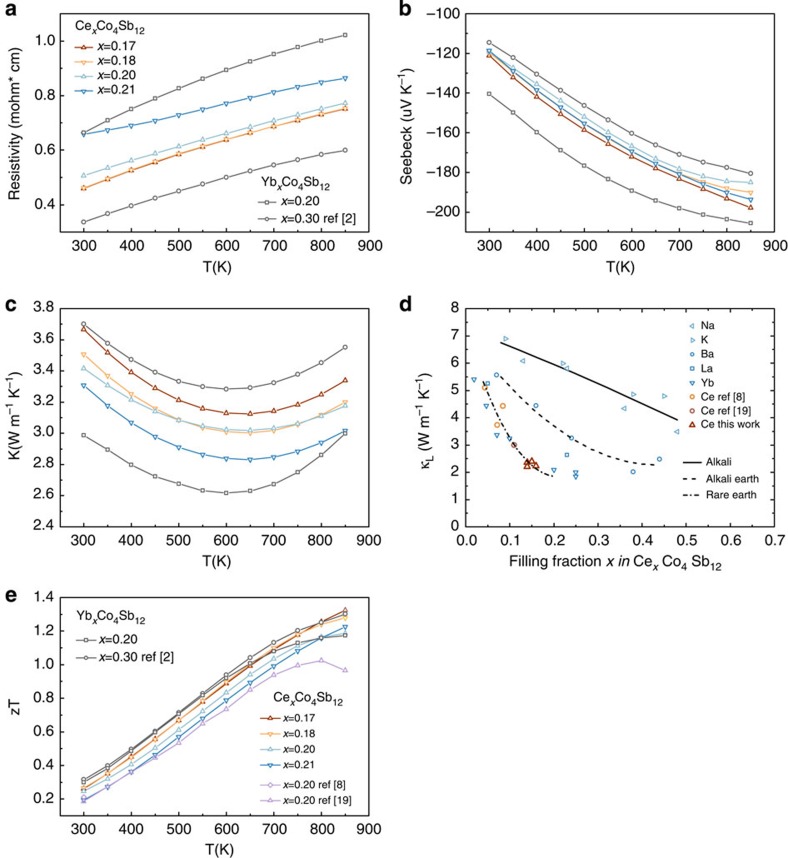
Transport properties of Ce- and Yb-doped skutterudites. The temperature dependence of: (**a**) electrical resistivity, (**b**) seebeck coefficient, (**c**) thermal conductivity, (**e**) thermoelectric figure of merit (*zT*) are plotted in the temperature range of 300–850 K. In figure (**d**) the lattice thermal conductivity with a Lorentz number of 2.0 × 10^−8^ V^−2^ K^−2^ is plotted against the filling fraction for various type of fillers. *x* denotes the nominal doping level of fillers. Yb_x_Co_4_Sb_12_ data for *x*=0.20 is from Tang, Y. *et al*. Convergence of multivalley bands as electronic origin of high thermoelectric performance in CoSb_3_ skutterudites, submitted.

Unfilled CoSb_3_ is an intrinsic semiconductor with carrier concentration <10^18^ cm^−3^. Rare earth filler atoms in the void sites are nearly perfect *n*-type donors. The *n*-type carrier concentration is essentially determined by the number of filling atoms and the ionic charge state. In CoSb_3_ a Yb filler has a valence charge +2 while Ce is +3, which is a lower oxidation state than often observed in oxides but common for Sb compounds^15^. Figure 1 demonstrates the trend of the carrier concentration versus the number of electrons per primitive cell, for either single, double or triple-filled skutterudites. To achieve high power factor of *n*-type skutterudites 0.4–0.6 electrons per Co_4_Sb_12_ unit is required, as indicated by the red-double arrow^16^. The carrier concentration of Ce-CoSb_3_ samples in the previous literature study^8^ is limited by the reported low filling fraction limit (*x*=0.09 in Ce_*x*_Co_4_Sb_12_ at 973 K), consequently these samples are far from the high-power factor region. To optimize the thermoelectric properties of Ce single-filled CoSb_3_ skutterudites, higher filling fraction of Ce is needed. As we shall show later, the filling fraction limit of Ce can be as high as *x*=0.20 at 1,123 K through solubility design, which is not only more than twice the reported value *x*=0.09 but now allows for the optimization of Ce-CoSb_3_. After optimization, the four samples in this work fall in the high power factor region (nominal Ce content, *x*=0.17, 0.18, 0.20, 0.21 and actual Ce content measured on hot pressed pellets are *x*=0.14, 0.14, 0.16, 0.15, respectively) and all of them have *zT* values reaching higher than 1.0 and above 750 K (see Fig. 2e).

### Thermoelectric properties of optimized Ce-CoSb_3_ skutterudite

**Figure 3:**
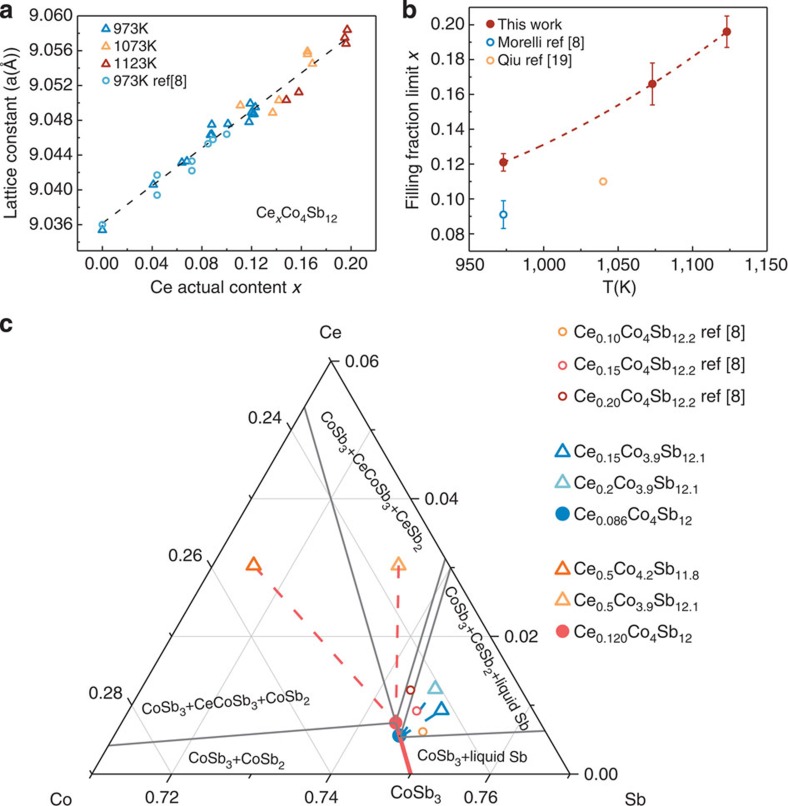
Filling fraction limit (FFL) of Ce in Ce-CoSb_3_ skutterudites. (**a**) Skutterudite lattice expansion due to Ce filling. (**b**) Dependence of FFL on annealing temperature. Red dashed line is a guide for the eye. Error bars represent the s.d. of filling fraction limit determined from EPMA measurements. (**c**) Dependence of FFL on nominal composition with annealing temperature 973 K.

The thermoelectric properties can be optimized when the filling fraction *x* of filler R (with valence *q*) in R_*x*_Co_4_Sb_12_ satisfies *qx*=0.4∼0.6 (refs 9, 16). From Fig. 3 we can see that when nominal compositions are in the Ce-rich three-phase regions with an annealing temperature at 1,073 and 1,123 K, the Ce actual content in skutterudite phase is about *x=*0.17 and 0.20, respectively. With Ce valence state being +3, *qx* for these materials would be in the optimum region. Thus, a set of samples with nominal compositions Ce_*x*_Co_4_Sb_12_ (*x=*0.17, 0.18, 0.20 and 0.21) were prepared for thermoelectric evaluation, of which the first two samples were annealed at 1,073 K and the latter two were annealed at 1,123 K. The temperature dependence of transport properties of these samples are shown in Fig. 2. Moreover, the transport data of two Yb-doped skutterudites with doping level *x=*0.20 and 0.30 are also plotted for the reason of comparison^2^ (Tang, Y. *et al.* Convergence of multivalley bands as electronic origin of high thermoelectric performance in CoSb_3_ skutterudites, submitted). With Yb valence state being +2, *qx* for these two samples are right on the boundary of the optimum region.

All the samples show negative Seebeck coefficients throughout the whole temperature range, indicating the expected *n*-type semiconductor behaviour. With increasing temperature, both the electrical resistivity and the magnitude of the Seebeck coefficient increase, which is typical behaviour for heavily doped semiconductors. It has to be noted that for the Ce-doped samples, the values of electrical resistivity, seebeck coefficient and thermal conductivity all fall between the values of the two Yb-doped samples, which is in agreement with Fig. 1. *κ*_*L*_ is obtained by subtracting the electronic contribution from the total thermal conductivity using the Wiedemann–Franz law. We adopted a Lorentz number of 2.0 × 10^−8^ V^−2^ K^−2^ to be consistent with previous work on skutterudites, and the lattice thermal conductivity calculated accordingly has <5% difference from the value calculated with the Lorentz number determined from a single parabolic band model^9^,^17^. All Ce-doped samples in this work show strongly reduced *κ*_*L*_ as compared with binary CoSb_3_ with *κ*_*L*_ ∼10 Wm^−1^ K^−1^ at 300 K. As apparent from Fig. 2d, Ce is as effective in reducing lattice thermal conductivity as Yb, with both much more effective than alkali and alkaline earth element fillers.

Figure 2e shows the continuously increasing *zT* indicative of the higher doping level than ref. 18. All Ce-CoSb_3_ samples in this work have *zT* values higher than 1.0 at and above 750 K, with the maximum *zT* value in Ce-doped skutterudite samples reaching 1.3 with nominal Ce content *x*=0.17 and 0.18 at 850 K (with composition Ce_0.14_Co_4_Sb_12_ for both samples determined from Electron Probe Micro-Analysis (EPMA)), which is 30% higher than that of previously reported literature value for Ce single-filled skutterudites (*zT*=1.0 for Ce_0.11_Co_4_Sb_12_ at 850 K)^18^. These *zT* are similar to those for single-filled skutterudites^2^,^19^,^20^,^21^, suggesting that Ce is a suitable earth abundant and low-cost replacement for other types of fillers, such as Yb, which makes the Ce single-filled skutterudites a more promising candidate for large-scale commercialization. The success of solubility design in Ce-CoSb_3_ system is a good example that this is an effective strategy in the optimization of thermoelectric properties in any other ternary systems with filling elements, which allows wide applicability of this strategy.

### Ultra-high filling fraction limit of Ce in Ce-CoSb_3_ skutterudites

The lattice expansion due to filling is an easy and effective way of characterizing the amount of fillers actually going into the void site in the skutterudite cell. Here lattice constants were derived from powder X-ray diffraction data and the actual Ce content was determined from EPMA. The lattice constant shows a simple linear dependence on the actual Ce content *x* in Ce_*x*_Co_4_Sb_12_, which agrees with the literature data and is consistent with Ce going into the same site across the whole range (up to *x*=0.20), presumably the void site. Samples annealed at higher temperatures show both higher Ce content by EPMA as well as higher lattice constants.

The partial filling of Ce in the void site increases the entropy due to the increase of disorder of the material, and thus it can be expected that the equilibrium solubility should increase measurably with temperature. While this is known theoretically^22^, the filling fraction limit is often considered to be a temperature-independent quantity; to date, there has been no report on the temperature dependence of filling fraction limit of Ce, let alone using this to design filler solubility in skutterudites. Here we show that the Ce filling fraction limit (determined from red point in Fig. 3c) increases significantly with temperature (see Fig. 3b and Supplementary Fig. 1). It increases to about 0.197
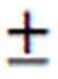
0.007 at 1,123 K and is more than twice the value reported from ref. 8 (*x*=0.09). This ultra-high filling fraction limit is verified using atom-probe tomography (APT) and is discussed later. This presents an opportunity for optimizing thermoelectric properties of Ce-CoSb_3_ skutterudites. The filling fraction limit *x*=0.120 at 973 K is also 25% higher than the reported literature value *x*=0.09, which can be explained due to the presence of separate three-phase regions in the Ce-Co-Sb system with different Ce solubilities in Ce_*x*_Co_4_Sb_12_.

Figure 3c shows a magnified region of an isothermal section at 973 K near CoSb_3_ of the Ce-Co-Sb ternary phase diagram. More details about phase diagram study are shown in Supplementary Figs 2–4 and Supplementary Table 1.

There are three two-phase regions and three three-phase regions near CoSb_3_. According to the phase rule, in any three-phase region, there are F=C-P+0=3−3=0 degrees of freedom where C is the number of components, P the number of phases and the 0 indicates both temperature and pressure are fixed. Thus, the compositions of the three equilibrium phases in a three-phase region are fixed and all the nominal compositions in this three-phase region will produce the same skutterudite composition with the same actual Ce content (marked as red and blue points in Fig. 3c), which are called stable skutterudite compositions in this study. This was confirmed by the EPMA measurements to be *x*=0.120±0.005 for the red stable composition (Ce_0.120_Co_4_Sb_12_) and *x*=0.086±0.007 for the blue stable composition (Ce_0.086_Co_4_Sb_12_) at 973 K.

When the nominal composition is slightly Co rich (in addition to sufficient Ce, for example, Ce_0.5_Co_4.2_Sb_11.8,_ red empty triangle) the sample is in the three-phase region between CoSb_2_, Ce-CoSb_3_ (ref. 23) and Ce_*x*_Co_4_Sb_12_ with *x*=0.120±0.005 (red point). Also if the nominal composition is slightly Sb rich (for example, Ce_0.5_Co_3.9_Sb_12.1,_ yellow empty triangle) the sample is in the three-phase region between CeSb_2_, Ce-CoSb_3_ and Ce_*x*_Co_4_Sb_12_ with, coincidentally, the same *x*=0.120±0.005 (red point) as found in the cobalt-rich three-phase region. In both cases the equilibrium Ce content in skutterudite phase will reach the same filling fraction limit *x*=0.120±0.005 at 973 K (red point) as indicated by tie lines (red-dashed lines). Note that these three-phase regions (one with CeSb_2_, Ce-CoSb_3_ and Ce_*x*_Co4Sb12; the other with CoSb_2_, Ce-CoSb_3_ and Ce*x*Co4Sb12) are different from the previous phase diagram study of Ce-Co-Sb system at 400 °C^23^. No similar ternary phase such as Ce-CoSb_3_ is found in the Yb-Co-Sb or In-Co-Sb system, but the Ga-Co-Sb system has a ternary phase (Co_3_Ga_2_Sb_4_) as well^24^.

However, when the nominal composition is very antimony rich, such as used by Morelli^8^ the samples are in another three-phase region between Ce_*x*_Co_4_Sb_12_, CeSb_2_ and liquid Sb (for example, Ce_0.2_Co_3.9_Sb_12.1_ and Ce_0.15_Co_3.9_Sb_12.1,_ marked as green and blue empty triangles, respectively). Here the Ce content in the skutterudite phase remains constant with *x*=0.087±0.007 (blue point) as indicated by tie lines (green-and blue-dashed lines). Note that two nominal compositions (Ce_0.10_Co_4_Sb_12.2_ and Ce_0.15_Co_4_Sb_12.2_) from ref. 8 fall into this three-phase region of Ce_*x*_Co_4_Sb_12_, CeSb_2_ and liquid Sb and one (Ce_0.20_Co_4_Sb_12.2_) falls into the two-phase region of Ce_*x*_Co_4_Sb_12_ and CeSb_2_. The observance of CeSb_2_ impurity phase in those samples confirms they are in a different field from that which gives the higher Ce content. The blue stable composition point coincides with the reported filling fraction limit from ref. 8, which is about 25% less of the filling fraction limit reported in this study for the same annealing temperature.

From the analysis of the temperature-dependent filling fraction limit we can also predict that the Ce content in the skutterudite phase can be controlled through the annealing temperature when the nominal composition is in one of the three-phase regions, which has proven to be a practical strategy to optimize thermoelectric performances.

**Figure 4:**
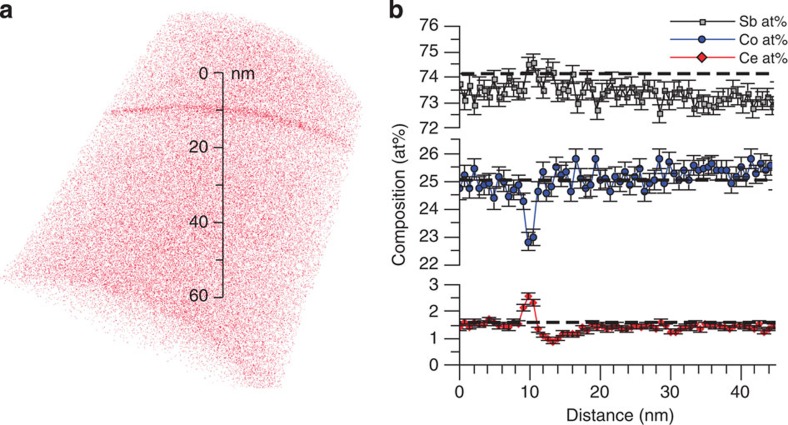
APT analysis of the most heavily doped sample Ce_0.2_Co_4_Sb_12_. (**a**) 3D reconstruction of microtip containing a grain boundary. Ce atoms are displayed in red; Sb and Co atoms omitted for clarity. (**b**) Concentration profile across the grain boundary and in the grain. The black dashed lines show values measured by EPMA, and the error bars represent the s.e., 
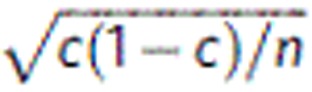
, where *c* is the concentration and *n* is the number of atoms detected in each data point.

APT measurements on Ce_*x*_Co_4_Sb_12_ skutterudite was also performed to confirm the ultra-high filling fraction of *x*=0.20. APT is particularly effective at chemically and structurally characterizing materials in 3D on the nanometre length scale. It provides accurate information about atomic concentrations and can identify even the smallest nanoparticles^25^. Small nanoparticles of Yb_2_O_3_ and InSb were observed in some Yb and In containing skutterudites, respectively^6^,^26^,^27^. Such small particles might go unnoticed in the Scanning Electron Microscopy (SEM) and would contribute to the EPMA signal. Here APT was used to verify the Ce content and homogeneity in the Ce_*x*_Co_4_Sb_12_ majority phase for sample annealed at 1,123 K with nominal composition Ce_0.5_Co_4_Sb_12_. According to EPMA, the Ce content in the majority skutterudite phase should be *x*=0.20 or 1.2 at%. Figure 4a contains a 3D reconstruction of the skutterudite phase at and around a grain boundary. As can be seen in Fig. 4b, atomic concentrations were consistent and homogeneous away from the grain boundary. No nano-precipitates of Ce-rich phases were observed. Atomic concentrations were averaged over a 30 nm diameter cylinder, from 20 to 60 nm on the scale bar in Fig. 4a, and the Ce content was measured to be 1.4±0.1 at%. This agrees with the values obtained by EPMA (*x*=0.20 corresponding to Ce content of 1.2 at %) and supports our conclusion that such a high filling fraction of Ce is indeed uniformly distributed in the skutterudite phase. A uniform accumulation of Ce at the grain boundary was also observed, accompanied by a reduction in Co. Such atomic segregation is frequently observed at grain boundaries^25^.

## Discussion

Because of the low cost of Ce as a single filler, Ce-CoSb_3_ skutterudites could have potential for scale-up commercialization in fields such as the automotive industry, if their thermoelectric performance can be optimized. Ce single-filled skutterudites in previous literature studies have been underdoped and fall out of the high power factor region due to the assumed low filling fraction limit of Ce (*x*=0.09). Here by conducting an equilibrium phase diagram study, we show that the filling fraction limit has a large temperature dependence and the value can reach as high as *x*=0.20 at 1,123 K. This is more than twice the value reported previously and thus allows to optimize Ce-CoSb_3_ skutterudites. This ultra-high Ce content is confirmed by APT measurements. The optimized Ce-CoSb_3_ skutterudites give a *zT* value of 1.3 at 850 K for Ce_0.14_Co_4_Sb_12_, which is among the highest reported *zT* values for single-filled skutterudites.

## Methods

### Sample preparation

High-purity elements Co (99.95%, slug), Sb (99.9999%, shot) and Ce (99.9%, rod) purchased from Alfa Aesar were used as raw materials. The samples were sealed in carbon-coated fused silica tubes under vacuum. The silica tubes were heated slowly up to 1,373 K in 12 h, held at this temperature for 12 h and then quenched in water to room temperature. Samples were then annealed at temperatures ranging from 973 to 1,123 K for 7 days. The resulting ingots were hand ground into fine powders and consolidated by rapid hot pressing at 973 K for 1 h under a pressure of about 60 MPa, yielding fully dense bulk samples. High density (>98% of the theoretical density of CoSb_3_) was achieved in all hot-pressed samples. Hot-pressed samples were sealed in fused tubes under vacuum for further annealing at the same annealing temperatures as before for 7 days again to erase the temperature effect of the hot pressing process before thermoelectric properties were measured^2^. Impurity phases were identified with X-ray diffraction and confirmed with SEM analysis in the same way as described in previous work^2^,^28^,^29^.

### Thermoelectric property measurements

Electrical transport properties, including electrical conductivity (*σ*) and Seebeck coefficient (*S*), were measured using the ZEM-3 (ULVAC.) apparatus under a helium atmosphere from 300 to 850 K. Thermal conductivity (*κ*) was calculated using *k*=*dD*_*T*_*C*_*P*_, with the thermal diffusivity *D*_*T*_ measured along the cross-plane direction by the laser flash method (Netzsch LFA 457) under argon flow with the Cowan model plus pulse correction. The density of the samples was measured using the geometrical method. The specific heat capacity *C*_*P*_ was determined using the Dulong—Petit law *C*_*P*_=3*k*_*B*_ per atom throughout the temperature range 300–850 K. The in-plane Hall coefficient (*R*_H_) was measured using the Van der Pauw method in a magnetic field up to 2 T^30^. Hall carrier concentration (*n*) was then estimated to be equal to 1/*R*_H_*e*, where *e* is the elementary charge. The Hall carrier mobility (*μ*_n_) was calculated according to the relation *μ*_n_=*R*_H_*σ*. The estimated measurement uncertainties are listed as follows: 5% for electrical resistivity, 7% for seebeck coefficient, 5% for thermal diffusivity and 1% for density. The data precision (reproducibility) is smaller than the accuracy (see Supplementary Fig. 5), leading to *zT* values within the range of ±0.2.

### Atomic probe topographic measurement

APT experiments were conducted on a Cameca LEAP-4000X Si equipped with a picosecond ultaviolet laser (wavelength 355 nm). Microtip samples of the nominal composition Ce_0.5_Co_4_Sb_12_ were prepared using a dual-beam focused–ion beam microscope (FEI Helios Nanolab) equipped with a micromanipulator (similar to the lift-out method)^31^. Microtips with a diameter of ∼100 nm were fabricated to contain a grain boundary and the last step of the tip sharpening process utilized a low voltage and current (5 kV, 16 pA) Ga^+^ ion beam to minimized Ga implantation in the sample (Ga content of the region analysed was <0.01 at%). The sample was maintained at 30 K and a laser energy of 10 pJ pulse^−1^ was used at a pulse rate of 250 Hz with a target evaporation rate of 0.5% atom pulse^−1^. The primary ions detected were Co^2+^, Co^+^, Ce^3+^, Ce^2+^, Sb^3+^, Sb^2+^ and Sb^+^. Very small amounts of Sb^2+^ and CoCe^+^, Ga^+^ and O^2+^ were also detected. Ions were detected using a two-dimensional microchannel plate detector with a detector efficiency of 50%. This detection efficiency is the same for all ions evaporated. The data collected were analysed and a three-dimensional reconstruction was created using the programme IVAS v.3.6.6.

## Additional information

**How to cite this article:** Tang, Y. *et al.* Solubility design leading to high figure of merit in low-cost Ce-CoSb_3_ skutterudites. *Nat. Commun.* 6:7584 doi: 10.1038/ncomms8584 (2015).

## Supplementary Material

Supplementary Information Supplementary Figures 1-5 and Supplementary Table 1
