# Supplementary material for: Solubility design leading to high figure of merit in low-cost Ce-CoSb3 skutterudites
Source: Nat Commun. 2015 Jul 20;6:7584. doi: 10.1038/ncomms8584 (PMC4518255; doi:10.1038/ncomms8584)
Supplement: Supplementary Information — Supplementary Figures 1-5 and Supplementary Table 1 [file ncomms8584-s1.pdf]

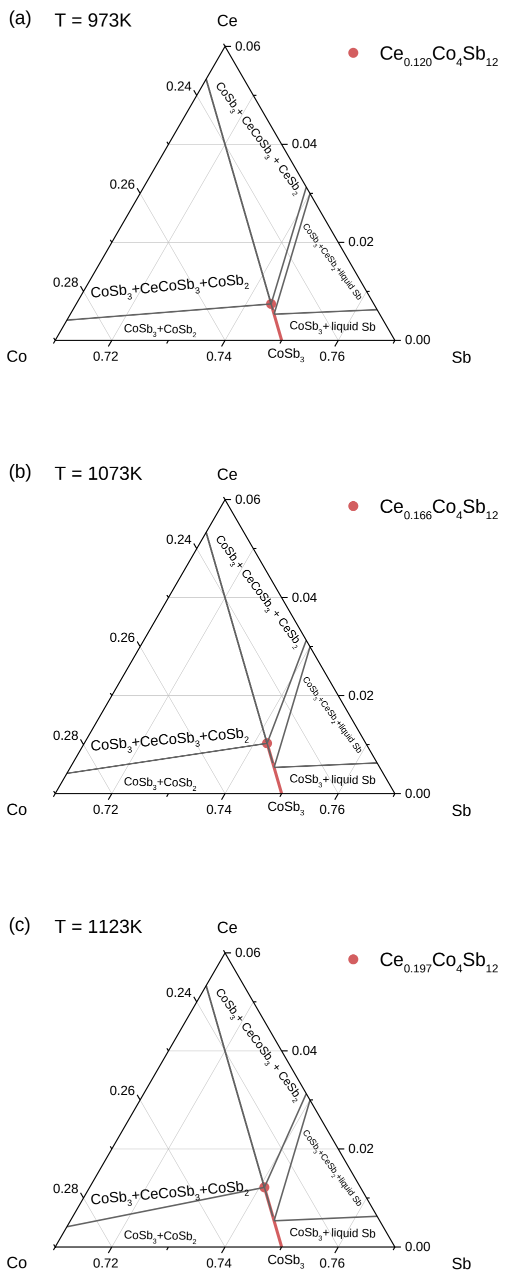

Supplementary Figure 1. Isothermal sections of Ce-Co-Sb phase diagram at various temperatures. The length of the red line represents the solubility of Ce in $CoSb_3$ skutterudite system which is determined from EPMA analysis. The point at which the three-phase region ($CoSb_3$ + $CeSb_2$ + liquied Sb) is in contact with the skutterudite phase region (red line) in both (b) and (c) is chosen to be the same as in (a) due to lack of further information.

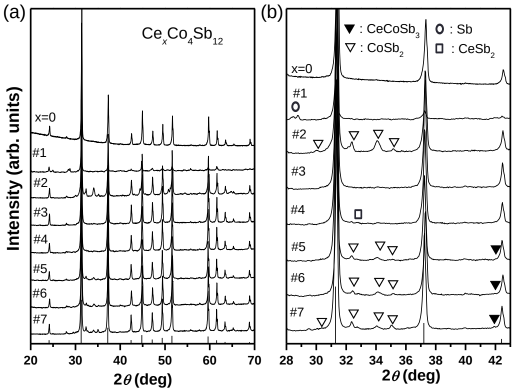

Supplementary Figure 2. X-ray diffraction (XRD) patterns. (a) XRD patterns of undoped $CoSb_3$ and the Ce-doped skutterudites annealed at 973K with nominal compositions #1 $Ce_{0.05}Co_{3.8}Sb_{12.2}$, #2 $Ce_{0.05}Co_{4.2}Sb_{11.8}$, #3 $Ce_{0.1}Co_4Sb_{12}$, #4 $Ce_{0.2}Co_{3.96}Sb_{12.04}$, #5 $Ce_{0.15}Co_4Sb_{12}$, #6 $Ce_{0.2}Co_4Sb_{12}$, and #7 $Ce_{0.3}Co_4Sb_{12}$. (b) Magnification of the XRD patterns in (a).

(a) $Ce_{0.05}Co_{3.8}Sb_{12.2}$

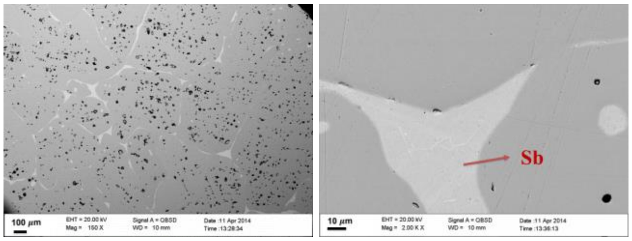

(b) $Ce_{0.05}Co_{4.2}Sb_{11.8}$

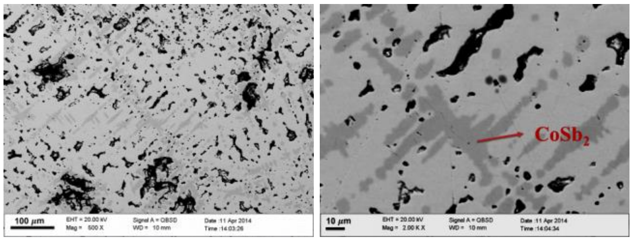

(c) $Ce_{0.1}Co_{4}Sb_{12}$

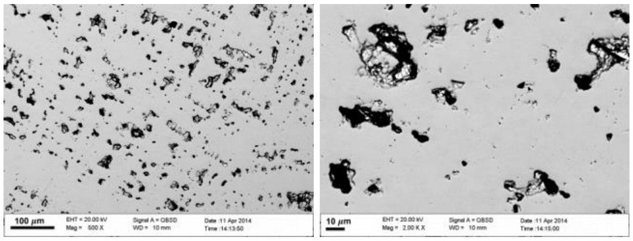

(d) $Ce_{0.2}Co_{3.96}Sb_{12.04}$

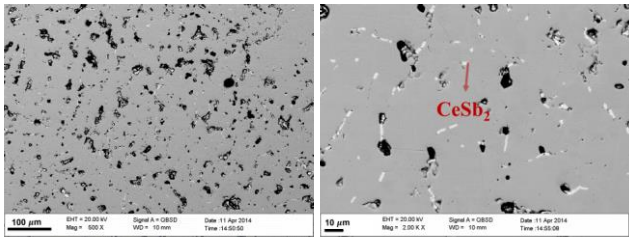

(e) $Ce_{0.5}Co_{4.2}Sb_{11.8}$

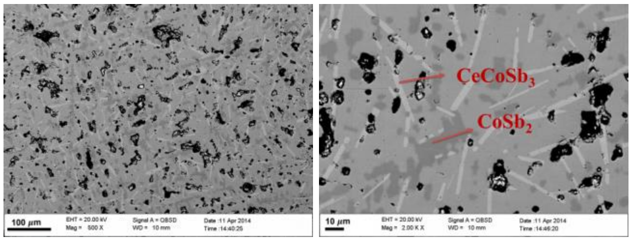

(f) $Ce_{0.5}Co_{3.9}Sb_{12.1}$

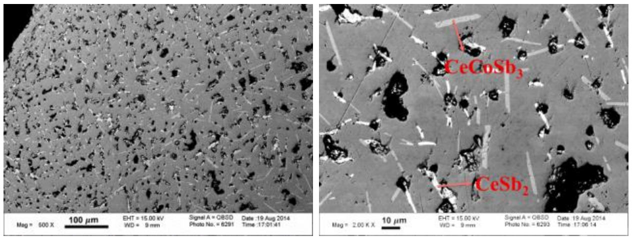

(g) $Ce_{0.3}Co_4Sb_{12}$

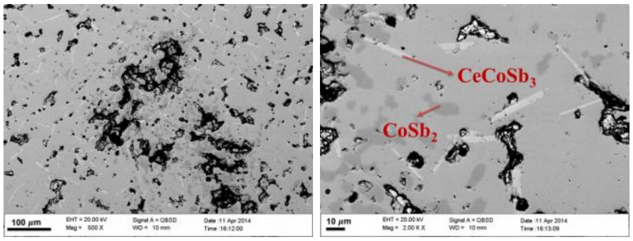

Supplementary Figure 3. Scanning electron microscope (SEM) images of samples annealed at 973K. Black regions are holes formed by thermal contraction during quenching. Scale bars are either in 100μm (left) or 10μm (right).

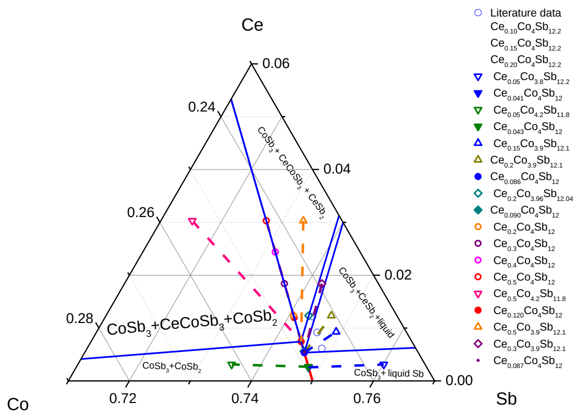

Supplementary Figure 4. Isothermal section of Ce-Co-Sb phase diagram at 973K with more sample details. A comparison between nominal compositions and actual EPMA compositions are shown more clearly in Supplementary Table 1 below.

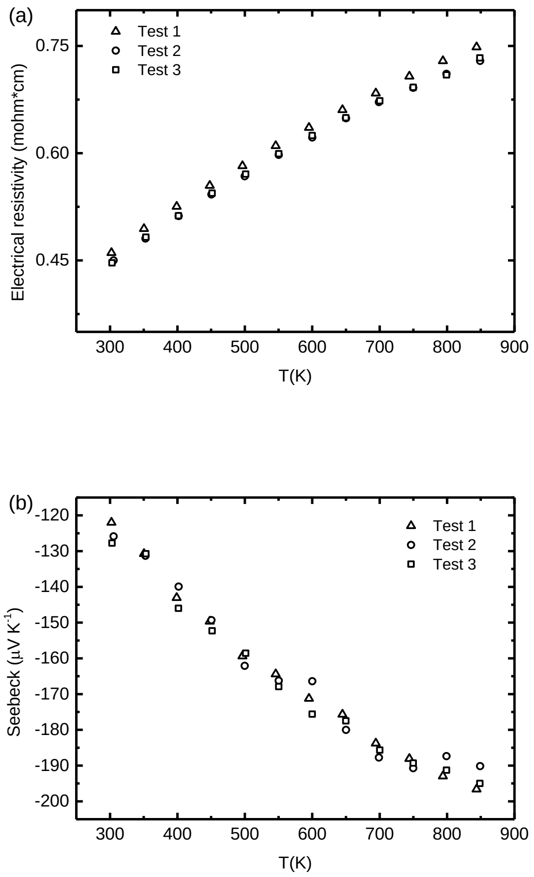

Supplementary Figure 5. Repeatability of thermoelectric properties of Ce-containing skutterudite $Ce_xCo_4Sb_{12}$ with doping level x = 0.14 higher than solubility limit at testing temperatures.

Supplementary Table 1. Actual Ce content in the skutterudite phase estimated by EPMA for Ce-containing skutterudites with different nominal compositions and annealing temperatures. Black, margenta and red boldface Ce contents correspond to the stable skutterudite composition represented as a red point in Supplementary Figure 1(a), (b) and (c) respectively. Blue Ce contents correspond to the stable skutterudite composition represented as a blue point in Figure 3(c).

Samples	Annealing T(K)	EPMA Ce content
$Ce_{0.05}Co_{3.8}Sb_{12.2}$	973	0.041 ± 0.009
$Ce_{0.05}Co_{4.2}Sb_{11.8}$	973	0.043 ± 0.005
$Ce_{0.05}Co_{4}Sb_{12}$	973	0.088 ± 0.006
$Ce_{0.5}Co_{4}Sb_{12}$	973	**0.120 ± 0.005**
	1073	**0.165 ± 0.013**
	1123	**0.197 ± 0.011**
$Ce_{0.5}Co_{4.2}Sb_{11.8}$	973	**0.121 ± 0.004**
	1073	**0.169 ± 0.010**
	1123	**0.195 ± 0.007**
$Ce_{0.4}Co_{4}Sb_{12}$	973	**0.122 ± 0.005**
	1073	**0.165 ± 0.013**
	1123	**0.196 ± 0.010**
$Ce_{0.3}Co_{4}Sb_{12}$	973	**0.121 ± 0.004**
$Ce_{0.2}Co_{4}Sb_{12}$	973	**0.119 ± 0.004**
$Ce_{0.5}Co_{4.05}Sb_{11.95}$	973	**0.118 ± 0.005**
$Ce_{0.5}Co_{3.995}Sb_{12.005}$	973	**0.123 ± 0.003**
$Ce_{0.5}Co_{3.95}Sb_{12.05}$	973	**0.122 ± 0.005**
$Ce_{0.5}Co_{3.9}Sb_{12.1}$	973	**0.120 ± 0.006**

$Ce_{0.2}Co_{3.96}Sb_{12.04}$	973	0.088 ± 0.05
$Ce_{0.15}Co_{3.9}Sb_{12.1}$	973	**0.080 ± 0.009**
	1073	0.097 ± 0.024
	1123	0.106 ± 0.017
$Ce_{0.2}Co_{3.9}Sb_{12.1}$	973	**0.090 ± 0.007**
	1073	0.111 ± 0.027
	1123	0.133 ± 0.014
$Ce_{0.3}Co_{3.9}Sb_{12.1}$	973	**0.087 ± 0.007**
